# Verrucous carcinoma of the esophagus with complete response after chemoradiotherapy

**DOI:** 10.1186/s40792-022-01486-7

**Published:** 2022-07-04

**Authors:** Masashi Hashimoto, Yasuhiro Shirakawa, Shunsuke Tanabe, Takehiro Tanaka, Naoaki Maeda, Kazufumi Sakurama, Kazuhiro Noma, Toshiyoshi Fujiwara

**Affiliations:** 1 Department of Gastroenterological Surgery, Okayama University Graduate School of Medicine, Dentistry and Pharmaceutical Sciences, 2-5-1 Shikata-cho, Kita-ku, Okayama, 700-8558 Japan; 2 Department of Surgery, Hiroshima City Hiroshima Citizens Hospital, 7-33 Motomachi, Naka-ku, Hiroshima, 730-8518 Japan; 3 Department of Pathology, Okayama University Graduate School of Medicine, Dentistry and Pharmaceutical Sciences, Okayama, Japan; 4 Department of Surgery, Shigei Medical Research Institute, Okayama, Japan

**Keywords:** Esophagectomy, Verrucous carcinoma, Esophageal squamous cell carcinoma

## Abstract

**Background:**

Verrucous carcinoma of the esophagus (VCE) is a rare tumor that is difficult to diagnose. In most cases, biopsies show nonspecific inflammatory and hyperkeratotic changes and do not show malignant findings. Most VCEs are slowly growing, locally advanced tumors with few metastases. Treatments for VCE are the same as for normal esophageal cancer, involving combined chemotherapy, surgical resection, and radiation therapy. However, it has been reported that VCE has a poor response to radiation or chemoradiotherapy (CRT). A case of VCE with complete response (CR) after CRT is presented.

**Case presentation:**

A 70-year-old man was found to have white, irregular esophageal mucosa 4 years earlier. He had been followed up as an outpatient as having candidal esophagitis. However, his tumor grew gradually, and biopsy was performed by endoscopic mucosal resection (EMR). He was finally diagnosed with VCE. He had no metastases to distant organs, but some lymph node metastases were suspected. The tumor invaded his left bronchus. The esophagostomy and gastrostomy were constructed as emergent procedures. The patient then underwent definitive CRT. 4 weeks after the end of CRT, two-stage esophagectomy was performed. First, he underwent esophagectomy with thoracic lymph node dissection. A latissimus dorsi flap was patched to the bronchus after primary suture of the hole. 6 weeks later, reconstruction of the gastric tube was performed through the antethoracic route. The pathological findings showed CR to CRT, with no proliferative cancer cells in the specimen. The patient has had no recurrence for three and half years after the resection.

**Conclusions:**

We presented a locally advanced VCE that achieved CR to CRT. In cases that have some difficulty for local resection, CRT might be an appropriate treatment for VCE.

## Background

Ackerman first reported verrucous carcinoma of the oral cavity in 1948 [1]. Although there have been some reports of verrucous carcinoma of various regions, reports of verrucous carcinoma of the esophagus (VCE) have been rare [2]. Minielly et al. reported the difficulty of making the diagnosis of VCE [3]. The findings of VCE on upper gastrointestinal endoscopy are a white, warty, plaque-like appearance with superimposed candidal esophagitis [2, 4]. The biopsies show nonspecific inflammatory and hyperkeratotic changes without malignant findings. Most cases of VCE are slowly growing, locally developing tumors with few lymph node or distant metastases [5]. However, the delay in making the diagnosis can sometimes be fatal [6]. The treatments for VCE are the same as those for common esophageal cancer, involving combined chemotherapy, surgical resection, and radiation therapy. Especially the far advanced esophageal cancer often is treated with radiation therapy. However, VCE has been reported to have a poor response to radiation or chemoradiotherapy (CRT) [5, 7]. Here, we present a rare case of locally advanced VCE with complete response (CR) after CRT with the pathological findings of a surgical specimen.

## Case presentation

**Fig. 1:**
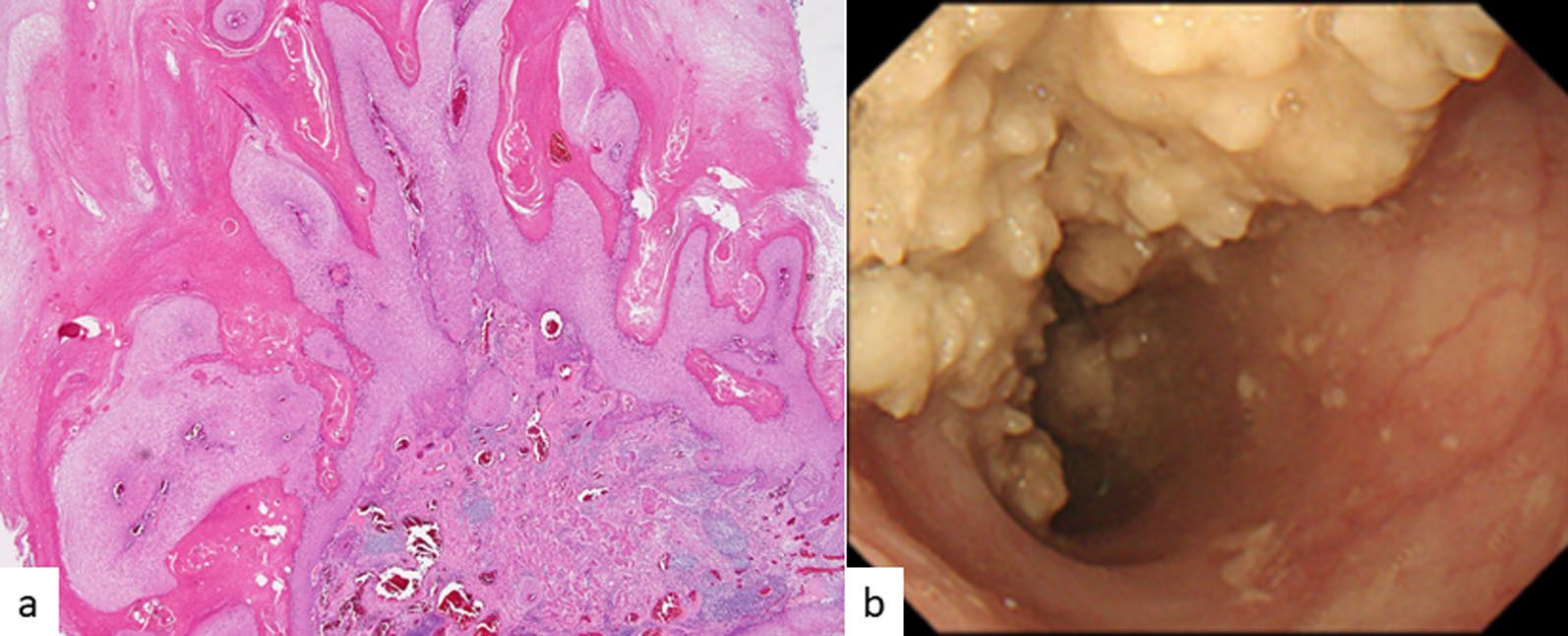
Images from gastrointestinal endoscopy and a histological biopsy specimen. **a** Hematoxylin and eosin staining shows malignant papillary carcinoma composed of keratinized and well-differentiated squamous cell carcinoma. **b** Gastrointestinal endoscopy shows a white, plaque-like, ulcerative appearance

**Fig. 2:**
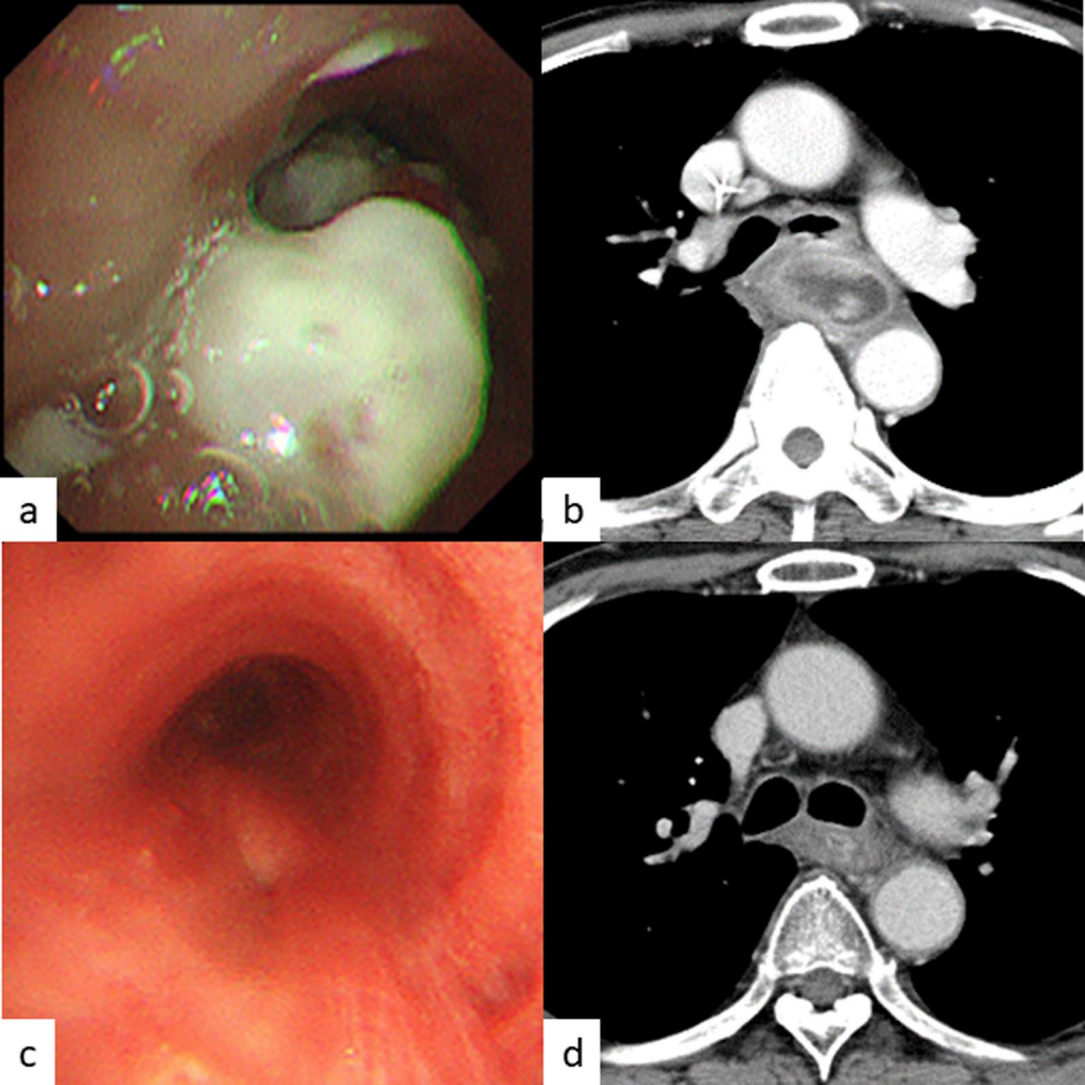
Images from bronchoscopy and CT. **a** Before CRT, bronchoscopy shows a white plaque-like erosion at the left bronchus. **b** Before CRT, CT shows the tumor invading the left bronchus. **c** After CRT, bronchoscopy shows a slightly depressed lesion. **d** After CRT, CT shows that the tumor has become smaller. There is no evidence of tumor invasion of the bronchus

A 70-year-old man was referred to our hospital complaining of obstruction that was due to type 3 VCE (MtLtUt, T4bN1M0, cStage IVA, UICC-8th) [8]. He had no history of smoking and drank alcohol occasionally. He had an upper gastrointestinal endoscopy every year. Although white, irregular esophageal mucosa was found 4 years earlier, there were no malignant cells in the first biopsy specimen, and candidal esophagitis was diagnosed. He had been followed up as an outpatient, but stenosis of the esophagus developed gradually. Taking into account his clinical course, endoscopic mucosal resection (EMR) was performed to obtain a large specimen for pathological examination. Histologically, the specimen was a malignant papillary tumor composed of markedly keratinized and well-differentiated squamous cell carcinoma (Fig. 1a). Based on these findings, the lesion was diagnosed as VCE. In our hospital, a further gastrointestinal endoscopic examination showed a tumor with a white, plaque-like, ulcerative appearance, 170 mm in diameter (Fig. 1b). Computed tomography (CT) and 8F-fluorodeoxyglucose (FDG) positron emission tomography (PET)/CT showed no distant metastases, but two regional lymph node metastases were suspected. Furthermore, CT and bronchoscopy showed that the tumor invaded the left bronchus and made an esophagobronchial fistula (Fig. 2a, b).

**Fig. 3:**
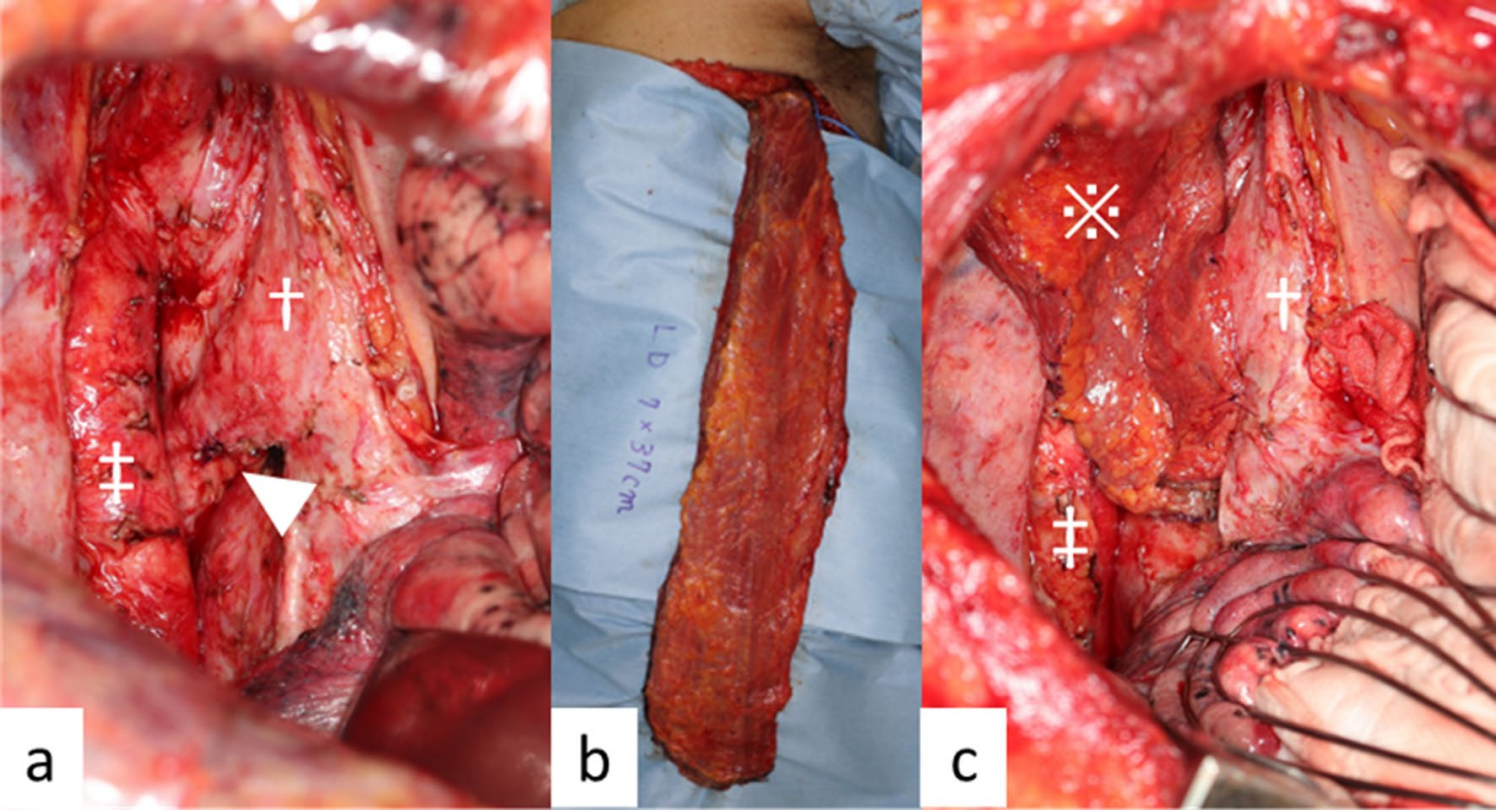
Surgical findings of esophagectomy. ※: latissimus dorsi flap, †: bronchus, ‡: descending aorta. **a** The hole of the left bronchus after esophagectomy is sutured (arrowhead). **b** Latissimus dorsi flap for patching the hole of the bronchus. **c** The latissimus dorsi flap has patched the hole of the left bronchus

**Fig. 4:**
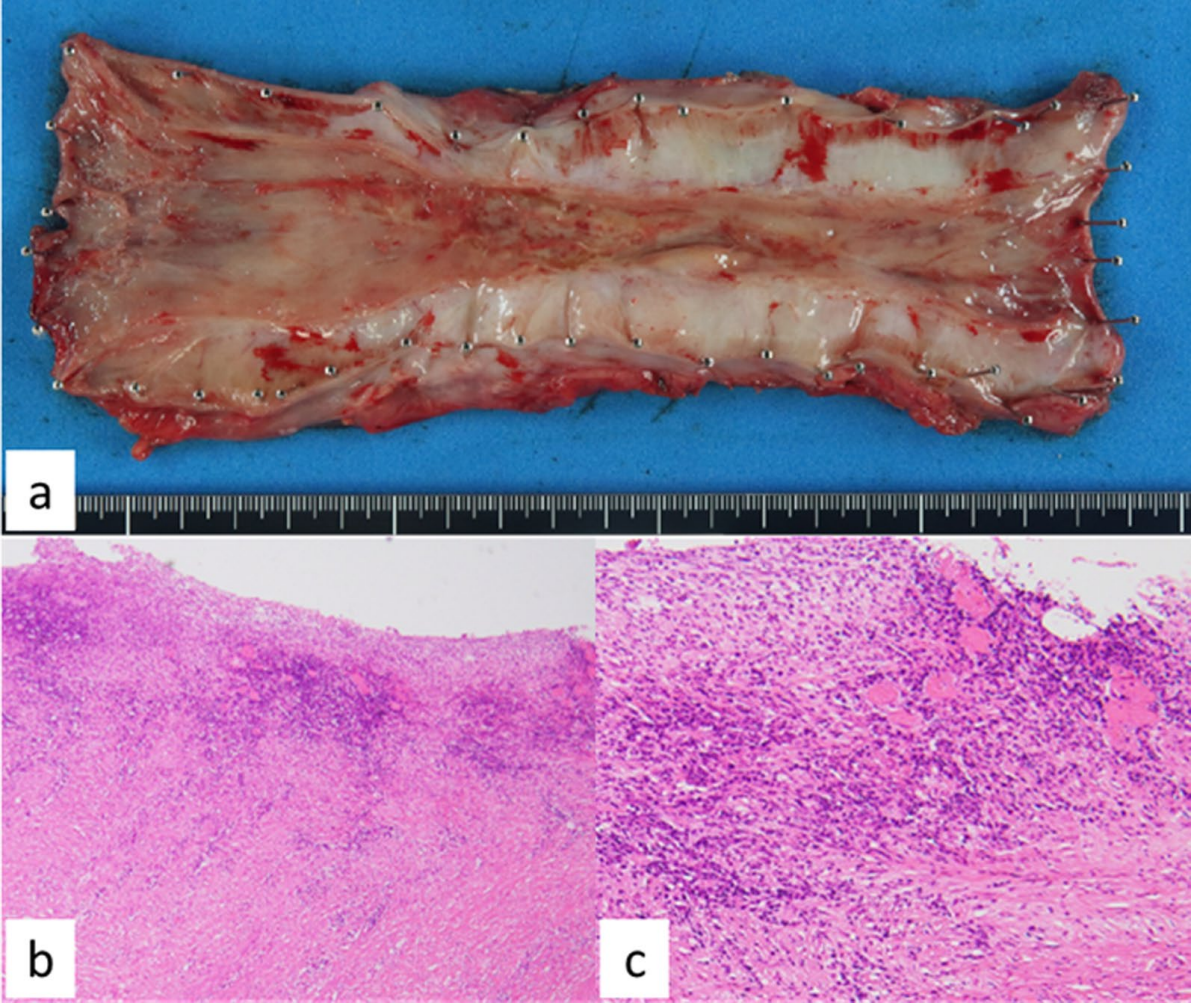
Resected specimen of the esophagus. **a** There is no apparent tumor on gross examination. **b**, **c** There are no malignant variable cells in the pathological findings, although inflammatory cells invade the superficial area. **c** Shows high-power magnification

The patient’s most important problem was that saliva entered his airway continuously, and this caused repeated aspiration pneumonia. This trouble prevented him from steady cancer treatments with esophagectomy or CRT. The cervical esophagostomy and percutaneous endoscopic gastrostomy (PEG) were performed as emergent procedures. The aim was to separate the flow of saliva from the airway. He then underwent definitive CRT (2 Gy/30 Fr, total: 60 Gy, 2 cycles of chemotherapy: 5-fluorouracil 700 mg/m^2^ and cisplatin 70 mg/m^2^ every 4 weeks), and this treatment was very effective. The tumor gradually decreased in size. Finally, an obvious esophagobronchial fistula developed at the membranous portion of the left bronchus (Fig. 2c). Re-examination of the tumor by upper gastrointestinal endoscopy could not be performed due to the esophagostomy. However, CT and FDG-PET–CT showed that the tumor had shrunk dramatically, and there were no new metastatic lesions (Fig. 2d). We decided to perform salvage surgery because the esophagobronchial fistula seemed unlikely to close with conservative treatment, and remaining tumor could not be completely ruled out from wall thickness. A two-stage operation was performed. In the first-stage surgery, esophagectomy with mediastinal lymph node dissection was performed. For the hole in the left bronchus due to the fistula (Fig. 3a), a latissimus dorsi flap (Fig. 3b) was inserted and patched at the dorsal side of the left bronchus after primary suture of the hole (Fig. 3c). Six weeks after first-stage surgery, second-stage surgery was performed with reconstruction with a gastric tube through the antethoracic route. The pathological findings showed CR to CRT, with no remaining malignant cells in the surgical specimen (Fig. 4a–c). The patient’s postoperative course was good. The patient was transferred to another hospital for rehabilitation 21 days after the second-stage operation. The patient has had no recurrence for three and half years after the resection.

## Discussion

A case of VCE with CR after CRT was reported. Since VCE was first reported in 1968 by Minielly, there have been more than 40 reports of it [3]. According to the World Health Organization classification, VCE is defined histologically as a malignant papillary tumor composed of well-differentiated and keratinized squamous epithelium [2]. In many reports, VCE was found to be associated with chronic mucosal irritation, achalasia, hiatal hernia, HPV infection, esophagitis, and so on. In the present case, there was no such history [5, 9], and the patient did not smoke and drank alcohol only occasionally.

The clinical and pathological diagnosis of VCE is difficult, as in verrucous carcinoma of other organs [4, 10]. Upper gastrointestinal endoscopy shows a white, warty, plaque-like appearance with superimposed candidal esophagitis [2] 4. Histologically, VCE shows a well-differentiated tumor or often a benign appearance. The superficial layer of VCE is covered with hyperkeratosis or acanthosis [4, 10]. The biopsy specimen from these layers showed no malignant diseases. The appearance often resembles that of candidal esophagitis [4]. The present case had been followed up as having candidal esophagitis for 4 years. Several biopsies did not show malignant cells. It was important to suspect that a case with such an appearance could be VCE. EMR was also useful method to obtain a large specimen for pathological examination [4].

Since VCE is a rare tumor, no standard treatment has been established. At present, multimodal therapy comprising surgery, endoscopic resection, chemotherapy, and/or radiotherapy (RT) is administered for VCE. Because of the features of local progression and rare lymph node metastases, endoscopic resection or esophagectomy has been commonly performed [5, 11]. Generally, RT is not the first-line treatment due to a report that verrucous carcinoma is less radiosensitive than the common type of squamous cell carcinoma (SCC) [7]. However, CRT was selected as the first-choice treatment strategy for the present case. Because of the tumor invasion to the left bronchus, it was judged that intensive preoperative therapy was indispensable for the esophagectomy without combined resection of the left trachea. Of course, verrucous carcinoma is less radiosensitive, but it is not radio-resistant [7, 12]. The details of the reason were unknown. Recently, the therapeutic outcomes of CRT for esophageal cancer have improved notably, and a better response might be expected than in the previous reports. We summarized 8 patients treated with CRT from five reports including our case [4, 13–15] (Table 1). Four cases underwent surgery after CRT: 2 patients achieved pCR, and 3 patients were alive for more than 3 years with no recurrence. The law of Bergonie and Tribondeau indicated the link between radiosensitivity and proliferation [16]. Verrucous carcinomas are highly differentiated tumors with low proliferation. However, most cancer has heterogeneity. Their clinical T stages were more than T2. Although the verrucous cell carcinoma covered the superficial region, the invasive region might include different radiosensitive cancer cells in good response cases. Fortunately, since the present case showed a good response to CRT, esophagectomy could be performed to treat the esophagobronchial fistula and the suspected remaining tumor. Furthermore, the pathological findings showed CR to CRT and, similar to other reports, no lymph node metastases. Surgical resection, the first choice for VCE, showed good outcomes in most cases. However, these results indicated that chemoradiation therapy could be another treatment option in the problematic cases of initial resection.

**Table 1:** Literature review of seven cases, including our case, of verrucous carcinoma of the esophagus treated with chemoradiation therapy

	Year	Gender/age	T	N	Chemotherapy	Surgery	Follow-up (years)	Outcome
Our case^b^	2022	M/70	4b	0	5-Fluorouracil + cisplatin	Yes	3	Alive
Al-Shoha et al. [13]^a^	2018	M/68	3	3	Unknown regimen	No	Unknown	Unknown
Ramini et al. [14]^a^	2014	M/78	4a	X	Unknown regimen	No	Unknown	Unknown
Sweetser et al. [4]	2014	M/61	3	0	Unknown regimen	Yes	6	Alive
	2014	F/73	Unknown	Unknown	Unknown regimen	No	3	Alive
	2014	F/63	3	0	Unknown regimen	No	1	Alive
	2014	M/68	2	0	Unknown regimen	Yes	2	Alive
Ahmed et al. [15]^b^	2013	F/58	3	X	Carboplatin + taxol	Yes	Unknown	Unknown

^a^Palliative CRT

^b^Pathological complete response

Due to a specific issue, the esophagobronchial fistula, the present case needed to undergo esophagectomy from the perspective of quality of life. However, it might be controversial whether to undertake surgery or not if without the fistula. However, the diagnosis of CR might be more difficult in this case of VCE because the diagnosis of CR is difficult even for squamous cell carcinoma, which was known to be radiosensitive [17]. Furthermore, stenosis often occurs too after CRT [11]. Although the response to CRT was good in the present case certainly, most cases reported previously were less radiosensitive. The present case must be followed up strictly and carefully for a long time if without surgery.

## Conclusions

We presented a locally advanced VCE that achieved CR to CRT. In cases that have some difficulty for local resection, CRT might be an appropriate treatment for VCE.

## Data Availability

Data sharing is not applicable to this article as no datasets were generated or analyzed during the current study.

## Abbreviations

VCE: Verrucous carcinoma of the esophagus
CRT: Chemoradiotherapy
CR: Complete response
Mt: Middle thoracic esophagus
Ut: Upper thoracic esophagus
Lt: Lower thoracic esophagus
EMR: Endoscopic mucosal resection
CT: Computed tomography
FDG: 8F-fluorodeoxyglucose
PET: Positron emission tomography
SCC: Squamous cell carcinoma
RT: Radiotherapy
